# Histoplasmosis infection in patients with rheumatoid arthritis, 1998-2009

**DOI:** 10.1186/1471-2334-11-145

**Published:** 2011-05-23

**Authors:** Timothy C Olson, Tim Bongartz, Cynthia S Crowson, Glenn D Roberts, Robert Orenstein, Eric L Matteson

**Affiliations:** 1 From the Department of Internal Medicine (TCO), Division of Rheumatology (TB, ELM, Division of Biomedical Statistics and Informatics (CSC), Department of Laboratory Medicine and Pathology (GDR), and Division of Infectious Diseases (RO), Mayo Clinic College of Medicine, Rochester, Minnesota, 55905, USA

**Keywords:** Histoplasmosis, rheumatoid arthritis

## Abstract

**Background:**

Patients with rheumatic diseases including rheumatoid arthritis (RA) are at increased risk for infections related to both the disease and its treatments. These include uncommonly reported infections due to histoplasmosis.

**Methods:**

Medical record review of all patients with a diagnosis of RA who developed new histoplasmosis infection in an endemic region between Jan 1, 1998 and Jan 30, 2009 and who were seen at Mayo Clinic in Rochester, Minnesota was performed.

**Results:**

Histoplasmosis was diagnosed in 26 patients. Most patients were on combination therapies; 15 were on anti-tumor necrosis factor (anti-TNF) agents, 15 on corticosteroids and 16 on methotrexate. Most received more than 6 months of itraconazole and/or amphotericin treatment. Two patients died of causes unrelated to histoplasmosis. Anti-TNF treatment was restarted in 4/15 patients, with recurrence of histoplasmosis in one.

**Conclusions:**

In this largest single center series of patients with RA and histoplasmosis in the era of immunomodulatory therapy, we found that most patients had longstanding disease and were on multiple immunomodulatory agents. Most cases were pulmonary; typical signs and symptoms of disease were frequently lacking.

## Background

*Histoplasma capsulatum *is a dimorphic fungus widely distributed in nature and is limited to the Midwest and Southeastern United States. After inhalation, the fungus transforms into a pathogenic yeast form. Optimal host defense against *Histoplasma capsulatum *requires interaction between macrophages and T-cells. Patients with rheumatic diseases receiving immunomodulatory and immunosuppressive therapies may be at increased risk for histoplasmosis, although reports of this infectious complication in this population primarily derived from case reports.

The most recent epidemiologic information for histoplasmosis was described in large urban outbreaks in Indianapolis that occurred between 1978 and 1993. The rate of dissemination of histoplasmosis from these studies has been cited at 0.46 per 1000 infected persons [1]. However immunosuppression has been a clearly identified risk factor in disseminated disease, and extrapulmonary disease occurs especially in immunocompromised patients [2]. Known immunosuppressive conditions noted to increase the risk of dissemination include acquired immunodeficiency syndrome, organ transplant, hematologic malignancies, and immunosuppressive agents [3].

Histoplasmosis occurs in patients with rheumatoid arthritis (RA) who receive corticosteroid therapy, disease modifying antirheumatic therapies such as methotrexate, and has been reported in post-marketing surveillance of patients receiving anti-tumor necrosis factor (TNF) therapy [4]. We describe the occurrence and risk factors for histoplasmosis in adult patients with RA in an endemic region in the era of anti-TNF therapies.

## Methods

We conducted a retrospective review of all patients with a diagnosis of RA who developed histoplasmosis and were seen at Mayo Clinic in Rochester, Minnesota between January 1, 1998 and January 30th, 2009. The Mayo Clinic Institutional Review Board approved the study.

The Mayo Clinic electronic medical record database was used to identify all adult patients (≥18 years of age) who were coded with the diagnosis of RA during the study period. All patients met the American College of Rheumatology classification criteria for RA [5]. Detailed information for each patient was abstracted from the medical record and included demographics, medical comorbidities including human immunodeficiency virus (HIV) and malignancy, date of diagnosis of RA, disease modifying antirheumatic drug (DMARD) therapies including biologic response modifiers and corticosteroid (CS) use, and the presence of coexisting infection at the time of diagnosis of histoplasmosis. Patient vital status and cause of death were recorded.

The Mayo Clinic Department of Laboratory Medicine and Pathology's clinical microbiology database was also used to screen and verify the diagnosis of histoplasmosis. Histoplasma infection was diagnosed by any of the following criteria: histopathology consistent with *Histoplasma capsulatum *by Gomori methenamine silver stain; positive culture of any tissue demonstrating *Histoplasma capsulatum*; a positive urine, serum or cerebrospinal fluid for *Histoplasma capsulatum *antigen by enzyme immunoassay screening; and Histoplasma antibody serology ≥1:8 done by complement fixation or a positive immunodiffusion test with the presence of M or H and M bands [6]. Supporting radiographic information included chest imaging (chest radiography or chest computed tomography scanning) at time of diagnosis.

All patients with RA and a verified diagnosis of active histoplasmosis were identified and included in the study analysis. Clinical signs and symptoms including fever, hepatosplenomegaly, respiratory, gastrointestinal, musculoskeletal, central nervous system findings, weight loss, lymphadenopathy and skin findings attributable to the histoplasmosis infection at time of infection were abstracted when available. The antifungal treatment history including timing and type of antifungal agent was retrieved. The DMARD regimen at time of Histoplasma infection and during treatment was recorded.

## Results

Twenty-six patients with a verified diagnosis of RA and meeting inclusion criteria for histoplasmosis were identified between January 1, 1998 and January 30, 2009. The characteristics of these patients are included in table 1. Fifteen patients were male and eleven were female. The mean age at the time of Histoplasma infection was 59.6 years (standard deviation, SD, 12.8), with mean duration of follow up of 2.1 years (SD 2.3). The mean duration of RA at the time of diagnosis of histoplasmosis was 10.5 years (SD 7.5). The most common comorbidity was chronic lung disease, found in 5 patients; and none had HIV or hepatitis B or C infection. Four patients had an underlying malignancy. Fifteen (58%) patients were hospitalized for diagnosis or management of their histoplasmosis, and 5 of these (19%) required initial treatment in a critical care unit. There were two deaths (8%) at time of last follow up. Neither death was as attributable to histoplasmosis.

**Table 1:** Summary of 26 patients with rheumatoid arthritis who developed histoplasmosis infection

Characteristic	Number (%) or years
Male	15 (58%)
Age, years (mean; range)	60 (32 - 78)
Duration of rheumatoid arthritis, years (mean; range)	10.5 (0.5 - 24)
Current disease modifying antirheumatic drug therapy	25 (96%)
Hospitalized for infection	15 (58%)
Duration of follow up, years (mean; range)	2.1 (0 - 7.9)
Patient deaths	2 (5%)
	
**Medication**	**Number (%)**
Methotrexate	21 (81%)
Prednisone (average dose: 9.1 mg)	15 (58%)
Hydroxychloroquine	5 (19%)
Leflunomide	5 (19%)
Anti-TNF Agent	15 (58%)
Adalimumab	7 (27%)
Infliximab	6 (23%)
Etanercept	2 (8%)
	
**Basis for Histoplasmosis Diagnosis**	**Number positive (%)**
Fever	19 (73%)
Abnormal chest imaging (X-ray or CT)	22 (85%)
Histoplasma serology	23 (88%)
Histoplasma culture	14 (54%)
Histoplasma urinary antigen	13 (50%)
Histoplasma biopsy histopathology	12 (46%)

Twenty-five of the 26 patients (96%) were on DMARD therapy with or without CS at the time of diagnosis of histoplasmosis. The most common DMARDs were methotrexate (81%) and anti-TNF agents (58%). Other therapies included hydroxychloroquine (19%) and leflunomide (19%). Fifty-eight percent of patients were on concurrent CS therapy (prednisone, average daily dose of 9.5 mg). Most (88%) were on combination therapy with DMARD or DMARD/CS at time of histoplasma diagnosis: 3 patients (12%) were on 4, 9 patients (35%) were on 3 and 11 patients (42%) were on 2 concomitant DMARDs (table 2). Anti-TNF agents included etanercept (2 patients; 8%), adalimumab (7 patients; 27%), and infliximab (6 patients; 23%). The immunomodulatory agents are summarized in table 1. The median time on anti-TNF therapy prior to histoplasma infection was 15 months (range: 2-132). The duration of treatment for each anti-TNF agent at the time of histoplasmosis is listed in table 2.

**Table 2:** Clinical characteristics and treatment of 26 patients with rheumatoid arthritis who developed histoplasmosis

Patient number	Age/Sex	Anti-TNF agent	Time to infection from initiation of anti-TNF agents (months)	Other therapy	Site of infection	Diagnosis*	Treatment
1	68/M	Adalimumab	48	Methotrexate, Prednisone	Lung	Serology	Itraconazole 3 months
2	60/M	Adalimumab	3	Hydroxychloroquine, Prednisone, Leflunomide	Lung	Serology	Itraconazole > 12 months
3	64/M	Adalimumab	2	None	Lung	Pathology (lung)	Amphotericin 2 weeks, voriconazole 3 months
4	62/M	Adalimumab	12	Methotrexate, Prednisone	Lung, liver	Culture (lung), pathology (lung), serology, urine antigen	Amphotericin 2 weeks, itraconazole > 12 months
5	47/F	Adalimumab	4	Methotrexate, Prednisone	Lung	Pathology (sputum), serology, urine antigen	Fluconazole 12 months
6	72/F	Adalimumab	15	Methotrexate	Lung, bone marrow, fungemia	Culture (sputum, bone marrow, blood), pathology (lung), serology, urine antigen	Amphotericin 2 weeks, voriconazole 10 weeks, itraconazole 12 months
7	47/F	Adalimumab	33	Methotrexate	Lung	Culture (sputum), serology, urine antigen	Itraconazole 16 weeks, transition to fluconazole 12 weeks (6 months total)
8	37/F	Etanercept	3	Methotrexate, Hydroxychloroquine	Lung	Serology	No treatment
9	47/F	Etanercept	2	Methotrexate	Lung	Serology	Itraconazole 1 week Voriconazole 12 months
10	59/M	Infliximab	12	Methotrexate, Prednisone, Leflunomide	Liver, fungemia	Culture (blood), pathology (liver), serology, urine antigen	Amphotericin 8 weeks, voriconazole 6 months transition to itraconazole > 12 months
11	73/F	Infliximab	24	Methotrexate, Prednisone	Bone marrow, fungemia	Culture (blood, bone marrow), serology	Itraconazole 3 months (follow up elsewhere)
12	32/M	Infliximab	18	Methotrexate, Hydroxychloroquine, Prednisone	Lung	Culture (sputum), pathology (lung), serology, urine antigen	Amphotericin 2 weeks, itraconazole > 12 months
13	68/F	Infliximab	36	Prednisone	Lung, small bowel	Culture (sputum, small bowel, peritoneum), pathology (small bowel) serology, urine antigen	Amphotericin 4 weeks, Itraconazole indefinitely
14	64/M	Infliximab	132	Azathioprine, Prednisone	Lung	Pathology (lung), serology	No treatment
15	53/F	Infliximab	56	Methotrexate	Lung	Serology, urine antigen	Voriconazole 1 week, Itraconazole 12 months
16	52/M	None	N/A	Methotrexate, Prednisone	Lung, bone marrow, fungemia	Culture (blood), pathology (bone marrow), serology, urine antigen	Amphotericin 1 week, Itraconazole 6 months
17	68/M	None	N/A	Methotrexate, Prednisone, Leflunomide	Lung	Serology	Itraconazole 5 months
18	68/M	None	N/A	Methotrexate, Prednisone	Lung, bone marrow	Culture (sputum), pathology (lung, bone marrow), serology, urine antigen	Itraconazole 12 months
19	70/M	None	N/A	Methotrexate, Prednisone, Leflunomide	Fungemia	Culture (blood)	Itraconazole 6 months
20	63/M	None	N/A	Methotrexate, Prednisone	Lung, Spleen	Culture (lung, sputum), serology, urine antigen	Itraconazole > 12 months
21	62/F	None	N/A	Methotrexate	Lung, fungemia	Culture (blood), serology	Amphotericin 2 weeks, itraconazole 12 months
22	78/M	None	N/A	Methotrexate, Prednisone	Lung	Culture (sputum), serology, urine antigen	Itraconazole 6 months
23	68/M	None	N/A	Methotrexate, Leflunomide, Minocycline	Lung, fungemia	Culture (blood), serology, urine antigen	Amphotericin 2 weeks, Itraconazole 12 months
24	35/F	None	N/A	None	Lung	Pathology (lung), serology	Itraconazole 3 months
25	75/M	None	N/A	Methotrexate, Hydroxychloroquine	Lung	Pathology (lung)	Itraconazole 6 weeks
26	45/F	None	N/A	Methotrexate, Hydroxychloroquine	Lung, liver	serology	Itraconazole 12 months

F = female; M = male; N/A = not applicable.

*See Methods for description of tests

The duration of symptoms prior to diagnosis of histoplasmosis was less than 4 weeks in 14 patients, 5 to 12 weeks in 10 patients and greater than 12 weeks in 2 patients. The lung was the site of infection in 14 patients. Nine patients had pulmonary disease and dissemination to an additional site including: liver or spleen involvement in 3 patients, fungemia in 2 patients, bone marrow involvement with fungemia in 2 patients, bone marrow involvement in one and small bowel involvement in one. The remaining 3 patients had disseminated disease without documented pulmonary involvement; one had fungemia, one had bone marrow involvement with fungemia and one had liver involvement with fungemia. Five patients had coexistent bacterial infections (*Staphylococcus aureus*, viridans group Streptococcus, Enterobacteriaceae, Bacteroides sp. and *Nocardia asteroides*).

Fever (>38°C) was the most common clinical manifestation of histoplasmosis at presentation (19 patients). Chest imaging (conventional radiograph or computed tomography) was obtained in 25 of 26 patients, including conventional radiograph in 24 patients at the time of diagnosis. The most common finding on plain chest radiograph was a focal pneumonitis in 9 patients, followed by an isolated pulmonary nodule in 5 patients. Computed tomography (CT) of the chest was obtained in 19 patients. The most common findings on CT were pulmonary nodules in 15 patients, hilar/mediastinal lymphadenopathy in 14 patients, and alveolar infiltrates in 7 patients.

Histoplasma antibody tests were obtained in all patients and were positive in 23 of the 26 patients. Histoplasma urinary antigen was obtained in 24 patients, and 13 were positive. Yeast cells of *Histoplasma capsulatum *were found in biopsies from 12 patients (8 lung tissue, 2 from bone marrow biopsy, and one each from liver and small bowel biopsy). Fungal cultures were obtained in 25 patients and 14 were positive; 8 had positive cultures from a pulmonary source (sputum, bronchial washings, and biopsy), 7 patients had positive blood cultures, 2 patients had positive bone marrow cultures and 1 had a positive small bowel tissue/peritoneal culture.

Antifungal therapy for histoplasmosis was administered to 24 of the 26 patients (92%). All treatment regimens included azoles. Itraconazole was used in 22, voriconazole in 4 and fluconazole in 2 patients. Nine of the 26 patients (35%) received an initial course of amphotericin (liposomal amphotericin), usually for 2 weeks in conjunction with an azole. The duration of antifungal therapy varied according to the clinical syndrome. Two patients with pulmonary histoplasmosis were untreated; the infection could be confirmed by biopsy in only one of them. The second untreated patient had a positive serology (H band only) and hilar lymphadenopathy on chest CT, and the diagnosis of acute histoplasmosis infection was in question.

Of 9 patients with pulmonary histoplasmosis and negative or unknown Histoplasma urinary antigen, 2 were treated for 12 months, and 5 were treated for less than 12 months. Of 5 patients with pulmonary histoplasmosis and a positive Histoplasma urinary antigen, one was treated for greater than 12 months, 2 were treated for 12 months and 2 patients were treated for 6 months. There were 12 patients with systemic histoplasmosis: one had lung and small bowel involvement and was treated indefinitely, 3 patients (lung with spleen, lung with liver, liver with fungemia) were treated for greater than 12 months, 5 patients (involvement of lung and bone marrow and fungemia in one; lung with fungemia in two, lung with liver in one, and lung with bone marrow in one) were treated for 12 months, and 3 patients (one with fungemia, one with lung, bone marrow and fungemia, and one with involvement of bone marrow with fungemia) were treated for less than 12 months.

Two of the patients died at time of last follow up. One patient had a pre-existent pulmonary adenocarcinoma. The second patient developed nocardiosis and herpes simplex virus infection approximately 12 months following completion of voriconazole therapy, and was treated for these but died 3 months after discharge of unknown causes.

Anti-TNF agents were discontinued in 14 of 15 patients taking these agents at time of diagnosis of the infection. The one patient who did not discontinue anti-TNF therapy had a lung biopsy to further assess pulmonary infiltrates. The biopsy revealed histoplasma granuloma but subsequent evaluation failed to reveal evidence of active disease. The diffuse lung infiltrates were felt to represent interstitial lung disease secondary to RA and not acute histoplasmosis. The patient's anti-TNF treatment (infliximab) was continued without interruption, and without development of overt histoplasmosis.

At the time of last follow up after histoplasma infection, 10 of the remaining 14 patients had not restarted anti-TNF agents. Four patients discontinued anti-TNF agents at the time of diagnosis and subsequently restarted them later (patients 1,3,8,12; table 2). Of these, three did not have a recurrence and one had a recurrence of clinical histoplasmosis **(**patient 12). This latter patient had been on infliximab for 18 months at the time of diagnosis of pulmonary histoplasmosis and was treated with itraconazole for 6 months with an appropriate clinical response. This was followed by re-introduction of adalimumab with continuation of itraconazole. The patient was hospitalized with recurrent fevers and an elevated histoplasma urinary antigen after 3 doses of adalimumab. A diagnosis of recurrent pulmonary histoplasmosis was made and the patient was treated with liposomal amphotericin for 2 weeks followed by itraconazole for a total of 2 years of therapy. The patient recovered from the histoplasmosis, and the anti-TNF therapy was not subsequently restarted.

The second of the three patients (patient 1 in table 2) whose anti-TNF therapy was resumed was diagnosed with pulmonary histoplasmosis with a positive serology and a pulmonary mass on CT with negative cultures and urinary antigen. The patient had been treated with adalimumab for 4 years prior to the diagnosis of histoplasmosis. Adalimumab was discontinued and the patient was treated with three months of itraconazole with improvement. Approximately one year after diagnosis of infection, the adalimumab was restarted. There was no recurrence of infection noted at time of last follow up.

The final of the three patients in whom anti-TNF therapy was resumed (patient 8) developed a febrile pulmonary illness three months after initiation of etanercept, which was then discontinued. The patient had hilar lymphadenopathy on chest CT and a low positive histoplasmosis serology (M band). The illness was self limited and improved on antibiotics without antifungal treatment. The etanercept was restarted 5 months after the resolution of the respiratory illness. There was no recurrence of pulmonary histoplasmosis in this patient at time of last follow up.

## Discussion

Histoplasmosis is endemic in the upper Midwest and Southeastern United States, and can occur in immunosuppressed patients as an opportunistic infection. It is infrequently reported in patients with RA. Immunomodulatory therapies, including anti-TNF agents and CS, pose a risk for development of histoplasmosis. The prevention and management of this disease in endemic areas poses significant challenges since most of the recommendations regarding diagnosis and management are based upon expert opinion [7]. Limited data suggests that serial monitoring with serial histoplasma serologies or urinary histoplasma antigen testing in a highly endemic area is not helpful to identify these patients before the onset of symptomatic disease [7]. Clinical decisions regarding discontinuing anti-TNF agents, use of preventive azole therapy and the timing of restarting immunosuppressive agents must be made in the face of considerable uncertainly. Most of the available clinical information on histoplasmosis in patients receiving anti-TNF agents is based upon case reports and small case series, with the largest report a literature review of fungal infections complicating anti-TNF therapy [8]. In that study, 84 cases of histoplasmosis were identified. In cases where data were available on the indication for anti-TNF therapy (42% of total cases), the majority of patients (77%) had RA.

The criteria for the diagnosis of histoplasmosis in our patient population is similar to other reported series. Histoplasma serologic tests were positive in 88% of our patients. This compares to previously reported sensitivities of this test in the 80% range [3,9]. Histoplasma urinary antigen was found to be positive in 54% of patients tested. In the subset of patients with disseminated disease 83% had a positive urinary antigen. The Histoplasma urinary antigen test is also useful for following the response to therapy, and assess for recrudescent infection [3]. CT and plain chest imaging was also helpful in the diagnosis of active infection; 88% of patients in this study had corroborative chest radiograph or CT findings. Culture and histopathology results remain important to identify organ involvement in disseminated disease, and were positive in 54% and 46% of our patients respectively.

Histoplasmosis in patients treated with immunomodulatory drugs including anti-TNF therapies may be the result of reactivation of latent infection, or new exposure (or reexposure) of hosts to the organism [10]. The risk of development of serious invasive fungal infections in patients on anti-TNF therapy has been increasingly recognized over the past several years [8,11,12]. Ten cases of histoplasmosis in patients taking anti-TNF agents from post licensure surveillance from the Adverse Event Reporting System (AERS) of the Food and Drug Administration (FDA) have been published [13]. In 2004, data from the AERS described an increased rate of granulomatous infections among patients on anti-TNF treatment [4]. In this series (1998 until 2002) there were 42 reported cases of histoplasmosis after the first anti-TNF treatments were approved for the treatment of rheumatoid arthritis in 1998. This association resulted in an FDA warning, issued on September 4, 2008, that notified healthcare professionals the recognition of histoplasmosis in patients taking anti-TNF agents may be associated with delays in treatment that can ultimately result in death [14]. At the time of this published warning, the FDA had reviewed 240 cases of histoplasmosis in patients who had received infliximab (207 cases), etanercept (17 cases), adalimumab (16 cases) and certolizumab (1 case). There were 12 deaths (5% of reviewed cases) noted in this report.

In the United States, the occurrence of tuberculosis in patients on anti-TNF therapy (most commonly in the first 90 days of treatment) supports reactivation of infection as the most likely mechanism of disease [4]. Reactivation and new infection are both possible in patients with RA treated with anti-TNF therapy [15]. The risk of reactivation histoplasmosis in 586 patients who underwent solid organ or bone marrow transplant in Indiana was reported to be very low [16]. Indiana is a hyperendemic area of histoplasmosis with 50-80% of residents having evidence of past infection as determined by skin testing. Despite the fact that 18% of patients had positive pretransplant histoplasmosis serologic tests and 4% had chest radiographs consistent with prior granulomatous disease, none developed histoplasmosis during a 3 year follow up time period. Serologic or radiographic evidence of prior histoplasmosis infection is not predictive of disease in the studies immunocompromised population.

In our population, the average time from initiation of anti-TNF therapy to clinical infection was > 15 months, making reactivation a less likely mode of acquisition of disease. There is no published evidence about whether granulomas on chest radiography in patients living in histoplasmosis endemic areas are predictive of development of histoplasmosis in patients on TNF-inhibitors. One patient in our study had biopsy proven pulmonary histoplasmosis. At that point the patient had been treated with anti-TNF therapy for 5 years without clinical evidence of disease. Another patient in our study had anti-TNF therapy restarted one year after treatment for symptomatic documented pulmonary histoplasmosis and had no evidence of reactivation of infection at time of last follow up one year after resumption of anti-TNF therapy.

The current study provides some assistance to clinicians regarding restarting immunomodulatory therapy in this group of patients. For the majority of patients on anti-TNF agents in our study, the therapy was discontinued at diagnosis and withheld throughout the period of histoplasmosis treatment. Patients in this study with disseminated disease were treated with azole therapy for 6 months or greater.

The most recent Infectious Disease Society of America clinical practice guidelines on the management of patients with histoplasmosis recommend 12 months of azole treatment for disseminated disease [17]. The prior guidelines however recommended 6-18 months of azole treatment for disseminated disease in non-AIDS patients [18]. The shorter treatment regimens in this patient population likely reflect treatment according to the earlier guideline. The treatment regimens in patients with acute pulmonary histoplasmosis were more variable, generally consisting of 3 months of azole treatment in less severe infection to 12 months or longer in more severe cases. The degree of immunodeficiency in RA patients also must be considered. Patients who require more intense immunosuppressive therapies for control of RA may even benefit from longer therapy or even lifelong suppressive therapy in select cases.

There are several limitations to this study. Although this is the largest single center experience with histoplasmosis in patients with RA in the era of biologic response modifiers, the number of patients is relatively small and conclusions regarding risk factors for infection or outcome must be drawn with caution. Confounding variables, the endemic nature of histoplasmosis, concomitant therapies and lack of epidemiologic information make attribution of specific therapy to histoplasmosis infection risk difficult. This was a single center review with a patient population primarily from Minnesota and neighboring states, the results of which may not be generalizable to other geographic areas. It is not a population based study, and we are unable to accurately identify a total number of patients exposed to each DMARD, including anti-TNF agents, to use as a denominator in the assessment of relative risk of developing histoplasmosis infection. Also, given the ubiquity of histoplasmosis in the environment, especially in the geographic area of this study, it is difficult to clearly identify mechanisms of exposure that resulted in disease.

How to screen for histoplasmosis and manage the patient with RA who has potential histoplasma exposure, lives in an endemic area for histoplasmosis, or has had previous overt histoplasmosis and requires immunomodulatory therapy, including anti-TNF treatment may be answered by further studies. At this point, we recommend that all patients in endemic areas be screened for a clinical history of histoplasmosis. Though chest radiography may not predict reactivation of histoplasmosis in patients on TNF-inhibitors, we believe it is valuable to determine pre-existing abnormalities in all patients in whom immunomodulatory therapy, including CS, conventional DMARD and biologic response modifier therapies. These studies are helpful to evaluate for the possibility of previous granulomatous infection, such as coccidiodomycosis and tuberculosis which may alter management strategies.

Screening with histoplasma urine antigen testing in the asymptomatic patient is appears to be of no benefit in predicting infection [7]. We have not found this to be a valuable screening tool and also do not recommend it. If there is evidence of previous exposure to histoplasma in the absence of previous overt infection, we do not recommend histoplasmosis prophylaxis. An important limitation of this recommendation is that the actual risk of reactivation of histoplasmosis is unknown, so that the risk/benefit of prophylactic treatment is also unclear.

Clinicians should proceed with caution when considering resumption of anti-TNF therapy in patients who clearly developed histoplasmosis infection while on anti-TNF agents. There is evidence that itraconazole maintenance therapy prevents relapse of histoplasmosis in AIDS patients who are chronically immunocompromised [19]. The risk of reactivation of severe histoplasmosis is unknown for any patient who was previously treated for the infection and who is then re-challenged with anti-TNF agents despite chronic azole therapy. Azole maintenance therapy may be effective in such patients who have reinstituted anti-TNF therapy [10].

## Conclusions

To our knowledge, this is the largest single center review of histoplasmosis in patients with RA in the era of anti-TNF agents. The patients who developed histoplasmosis had longstanding RA (greater than 10 years) and were treated with multiple immunomodulatory agents, with 88% of patients on more than one agent at time of diagnosis. More than 50% of RA patients who developed histoplasmosis during the 11 year study period were on anti-TNF treatment. Infection can occur at any time after initiation of anti-TNF therapy, so that a high index of suspicion is required for febrile patients with pulmonary symptoms. Histoplasma serologic tests were frequently positive in this patient population and remain useful diagnostic tools. Forty six percent of patients in our study had severe disseminated, extrapulmonary disease.

Prolonged treatment regimens with azole therapy are often required to resolve the disease. It is prudent to discontinue anti-TNF agents at time of infection. Resumption of immunomodulatory therapies including anti-TNF therapy must be undertaken cautiously in patients who developed histoplasmosis while on anti-TNF therapy as there is a risk of serious relapse of the infection despite suppressive antifungal therapy. The majority of cases may be secondary to reinfection rather than reactivation. There are no accepted screening strategies to assess patients at risk for infection, so that clinicians and patients must maintain vigilance for the possibility of development of active histoplasmosis.

## Competing interests

All authors declare that they have no competing interests with respect to this manuscript. Funding for costs related to this study, including statistical analysis and article processing charges, was provided by the Mayo Foundation.

## Authors' contributions

TCO participated in study design, data acquisition, data analysis and interpretation, and manuscript preparation. TB participated in study design, data acquisition, data analysis and interpretation and manuscript preparation. CSC participated in study design, data analysis and interpretation and manuscript preparation. GDR participated in data acquisition and data analysis and interpretation and manuscript preparation. RO participated in study design, data acquisition, data analysis and interpretation and manuscript preparation. ELM participated in study design, data acquisition, data analysis and interpretation and manuscript preparation. All authors read and approved the final manuscript.

## Pre-publication history

The pre-publication history for this paper can be accessed here:

http://www.biomedcentral.com/1471-2334/11/145/prepub
